# The many faces of diabetes. Is there a need for re-classification? A narrative review

**DOI:** 10.1186/s12902-021-00927-y

**Published:** 2022-01-07

**Authors:** Nasser Sakran, Yitka Graham, Tadeja Pintar, Wah Yang, Radwan Kassir, Edith M. Willigendael, Rishi Singhal, Zoë E. Kooreman, Dharmanand Ramnarain, Kamal Mahawar, Chetan Parmar, Brijesh Madhok, Sjaak Pouwels

**Affiliations:** 1 Department of Surgery, Holy Family Hospital, Nazareth, Israel; 2 the Azrieli Faculty of Medicine, Bar-Ilan University, Safed, Israel; 3 Faculty of Health Sciences and Wellbeing, University of Sunderland, Sunderland, UK; 4 Facultad de Psycologia, Universidad Anahuac Mexico, Mexico City, Mexico; 5 Department of Abdominal Surgery, University Medical Center Ljubljana, Zaloška cesta, Ljubljana, Slovenia; 6 Department of Metabolic and Bariatric Surgery, The First Affiliated Hospital of Jinan University, 613 Huangpu Avenue West, Guangzhou, Guangdong Province China; 7 CHU Félix Guyon, Allée des Topazes, Saint-Denis, France; 8 Department of Vascular Surgery, Medisch Spectrum Twente, Enschede, The Netherlands; 9 Bariatric and Upper GI Unit, Birmingham Heartlands Hospital, University Hospital Birmingham NHS Foundation Trust, Birmingham, UK; 10 Department of Dermatology, Amphia Hospital, Breda, The Netherlands; 11 Department of Intensive Care Medicine, Elisabeth-Tweesteden Hospital, Tilburg, The Netherlands; 12 Bariatric Unit, South Tyneside and Sunderland NHS Foundation Trust, Sunderland, UK; 13 Department of Surgery, Whittington Health NHS Trust, London, UK; 14 East Midlands Bariatric and Metabolic Institute, University Hospital of Derby and Burton NHS Foundation Trust, Burton, UK; 15 Department of Intensive Care Medicine, ETZ Elisabeth, Hilvarenbeekseweg 60, P.O. Box 90151, 5000 LC Tilburg, The Netherlands

**Keywords:** Diabetes mellitus, Bariatric surgery, Antidiabetic drugs, Gastrointestinal hormones, Metabolic surgery, Classification

## Abstract

The alarming rise in the worldwide prevalence of obesity and associated type 2 diabetes mellitus (T2DM) have reached epidemic portions. Diabetes in its many forms and T2DM have different physiological backgrounds and are difficult to classify. Bariatric surgery (BS) is considered the most effective treatment for obesity in terms of weight loss and comorbidity resolution, improves diabetes, and has been proven superior to medical management for the treatment of diabetes. The term *metabolic surgery* (MS) describes bariatric surgical procedures used primarily to treat T2DM and related metabolic conditions. MS is the most effective means of obtaining substantial and durable weight loss in individuals with obesity. Originally, BS was used as an alternative weight-loss therapy for patients with severe obesity, but clinical data revealed its metabolic benefits in patients with T2DM. MS is more effective than lifestyle or medical management in achieving glycaemic control, sustained weight loss, and reducing diabetes comorbidities. New guidelines for T2DM expand the use of MS to patients with a lower body mass index.

Evidence has shown that endocrine changes resulting from BS translate into metabolic benefits that improve the comorbid conditions associated with obesity, such as hypertension, dyslipidemia, and T2DM. Other changes include bacterial flora rearrangement, bile acids secretion, and adipose tissue effect.

This review aims to examine the physiological mechanisms in diabetes, risks for complications, the effects of bariatric and metabolic surgery and will shed light on whether diabetes should be reclassified.

## Background

As far back as 1550, Before Common Era (BCE), history documents words on an Egyptian papyrus referring to an illness in which patients lost weight and urinated frequently. Over 1000 years later, Appolonius was credited with being the first to use the word ‘diabetes,’ with Galen suggesting diabetes is kidney disease [1]. In 5 Common Era (CE), Sushruta, the Indian surgeon, noted the sweetness of the urine, it is sticky feeling, and the ability to attract ants, and noted that diabetes tended to afflict the higher castes and related the condition to excessive consumption of rice, cereal, and sweet foods [2]. In the late 1700s, Dobson discovered that the sweet taste in the urine of people with diabetes is due to excess sugar in both urine and blood. His observations of patients lead to future differentiation of Type I and Type 2 Diabetes (T2DM) [3]. In 1889, Mering and Minkowski discovered that removing the pancreas from dogs led to developing diabetes and death helped scientists understand the association with pancreas and blood sugar levels. Soon after, Sharpey-Schafer posited that diabetes developed due to a lack of a substance in the pancreas, which he called insulin [4]. The discovery of insulin by Banting and Best in 1921 heralded a breakthrough for diabetes treatment, with the first human to receive insulin treatment in 1922. In 1936, Himsworth differentiated between Type 1 and T2DM, suggesting insulin resistance rather than deficiency [4]. Rates of diabetes declined in Germany and other European countries during WWI and World War II, attributed to food shortages; this was not apparent in Japan and North America, where there was no rationing [5]. Table 1 gives an overview of the pivotal historical events in recognizing diabetes as a disease and the development of early diagnostic and therapeutic measures.

**Table 1:** A brief history of diabetes mellitus research and development [6–8]

Time	Development
	Variations of diets
**550 Common Era (CE)**	Bleeding, emetics and narcotics
**1921**	Discovery of insulin
**1923**	Commercial animal insulin available
**1936**	Introduction of long-acting insulin
**1941**	Urine-testing tablets for sugar available
**1949**	Syringe drivers for insulin
**1961**	Glucagon available for hypoglycemia
**1971**	The first blood glucose meter developed
**1972**	Metformin approved for use in Canada
**1983**	Recombinant insulin developed
**1995**	First Alpha-glucosidase inhibitor (AGI) Acarbose
**1996**	First Thiazolidinedionederivate (TZD) Troglitazone
**1997**	First Meglitinide Repaglinide
**2005**	First Amylin agonist pramlintide First Glucagon-Like Peptide 1(GLP-1) receptor agonist (exenatide)
**2006**	First Dipeptidyl peptidase-4 (DPP-4) inhibitor (sitagliptin)
**2008**	Colesevelam approved for diabetes
**2009**	Bromocriptine approved for diabetes
**2013**	First Sodium Glucose Transporter-2 (SGLT-2) inhibitor (canagliflozin)

With the rise of bariatric and metabolic surgery (BMS), new surgical and physiological challenges became apparent. Evidence has shown that endocrine changes resulting from surgery translate into metabolic benefits that improve the comorbid conditions associated with obesity, such as hypertension, dyslipidemia, and T2DM. Other changes include bacterial flora rearrangement, bile acids secretion, and adipose tissue effect. This review aims to examine the physiological mechanisms in diabetes, risks for complications, the effects of BMS and will shed light on whether diabetes should be reclassified.

## Pathophysiology and definition of diabetes in general

The pathophysiology and definitions of diabetes are complex, and with hormonal discoveries, it gets even more complex in the last few years. In general, diabetes can be classified into the following categories [9, 10]: Type 1 Diabetes Mellitus (T1DM) (due to beta cell destruction, nearly always insulin deficient); Type 2 diabetes (T2DM) (due to a progressive insulin secretory defect on the background of insulin resistance). Within this spectrum there are several types: Gestational diabetes mellitus (GDM) (diabetes diagnosed in the second or third trimester of pregnancy that is not overt diabetes) and specific types of diabetes due to other causes, e.g., monogenic diabetes syndromes (such as neonatal diabetes and maturity-onset diabetes of the young [MODY]), diseases of the exocrine pancreas (such as cystic fibrosis), and drug- or chemical-induced diabetes (such as in the treatment of HIV/AIDS or after organ transplantation) [9, 10].

T1DM is considered a chronic autoimmune disorder caused by the progressive T-cell mediated destruction of pancreatic β-cells that produce insulin. Apart from the decline in β-cell mass due to this phenomenon, the gradual loss of glucose sensitivity of the β-cells contributes to the deficient levels of insulin, causing hyperglycemia [6, 9, 11]. T1DM frequently occurs in childhood and has a peak incidence at 10–14 years [12]. The pathogenesis of T1DM consists of a complex interplay between a predisposing genetic risk and environmental factors and triggers [13]. The primary risk factor for β-cells auto-immunity is genetic, mainly in individuals with one or both human leukocyte antigen (HLA) class 2 haplotypes involved in antigen presentation. Studies in first-degree relatives show that the presence of two or more β-cell targeting autoantibodies is a predictor for T1DM. The disease progression depends on the age of antibody detection, amount of antibodies; its specificity, and antibody titer [6, 9, 10]. Environmental factors that trigger auto-immunity are infections, dietary factors, psychosocial stress, and altered intestinal microbiome composition [14].

Altered gut microbiome trigger host metabolism via modulating the production of short chain fatty acids (SCFA) in T2DM and is related to insulin sensitivity, lipid and glucose metabolism. Intestinal dysbiosis is related to increased intestinal permeability that allows translocation of bacterial lipopolysaccharide into systemic circulation. As a consequence, liver fat accomulation progresively increase fibrosis, weight gain and progression of T2DM. Furthermore, intestinal microbiome may have suppresive effect on fasting-induced adipocyte factor which acts as an inhibitor to circuating lipoprotein lypase and this consequently increas triacylglycerol (TAG) storage in periferal tissues. Microbiota also, is responsable for metabolism of liver derivated primary bile acids into.

secondary bile acids are reabsorbed into systemic circulation and take over the function of signaling molecules (interplayed via farnesoid receptor X, FXR) included in glucose homeostasis regulation [14].

Over 90% of all diabetes are cases of T2DM, a chronic metabolic disease characterized by a relative insulin deficiency due to the combination of deficient secretion, tissue insulin resistance, and inadequate compensatory mechanisms. In a later stage, unsustainable serum glucose levels can lead to diabetic complications [15]. In an excessive nutritional state, hyperglycemia and hyperlipidemia can ultimately cause inflammation and stress on the β-cells, leading to dysfunction and further stage atrophy. At diagnosis, up to 50%, β-cell loss is described. Insulin resistance causes an increased glucose production in the liver and reduces peripheral glucose uptake in the muscle, liver, and adipose tissue [16]. Hypertrophic adipose tissue in obesity stimulates insulin resistance through increased circulation of pro-inflammatory and free fatty acid release. The chronic mild inflammatory state represents a key part of the pathogenesis of T2DM [15].

## Complications of diabetes mellitus

Diabetes is a disease that is strongly associated with both macrovascular complications, including ischemic heart disease, cerebrovascular disease, and peripheral vascular disease, and microvascular complications, including nephropathy, retinopathy, and neuropathy. This results in organ and tissue damage in approximately one-third to one-half of patients with diabetes [17]. Diabetes-associated vascular alterations include anatomic, structural, and functional changes leading to multi-organ dysfunction [18].

The relationship between poor glycaemic control and microvascular and macrovascular complications was established in the prospective Diabetes Control and Complications Trial (DCCT) [19–21]. The cause for the increased morbidity and mortality is a direct result of diabetes and is a consequence of the combination of macrovascular (atherosclerosis) and microvascular disease. The importance of tight glycemic control for protection against macrovascular disease in diabetes has also been established in the DCCT/Epidemiology of Diabetes Interventions and Complications (EDIC) study for T1DM [22, 23].

For patients with T2DM, only limited clinical trial data have shown a macrovascular benefit with intensive therapy [24]. Selecting appropriate target glycated hemoglobin (A1C) should be individualized based upon individual comorbidities and functional status. The results of the Action to Control Cardiovascular Risk in Diabetes (ACCORD) trial suggest that a target A1C of 7.0 to 7.9% may be safer for patients with longstanding T2DM and who are at high risk for cardiovascular disease (CVD) than a target A1C of less than 6.0% [25]. With respect to patients with newly diagnosed T2DM, the literature shows a long-term improvement in reducing the risk of myocardial infarction, diabetes-related death, and overall death after an intensive control (A1C 7%) [26, 27]. Improved glycaemic control lowers the risk of microvascular complications in patients with type 2 diabetes [24, 25, 28–31]. However, the absolute risk for microvascular complications and the incremental benefit of intensively lowering A1C must be balanced against the diminishing returns and the heightened risk of hypoglycemia at A1C levels less than 6.5%. The A1C goal should be set somewhat higher (< 8% or higher) for patients with a history of severe hypoglycemia, patients with limited life expectancy, very young children or older adults, and individuals with advanced complications or comorbid conditions [7, 32].

## Macrovascular complications

Cardiovascular disease (CVD) causes up to 65% of all deaths in people with diabetes [33]. For patients with T2DM, CVD is the leading cause (70%) of death [33]. Diabetes gives a 4-fold-greater risk for CVD’s compared to patients without diabetes, corrected for risk factors like age, obesity, tobacco use, dyslipidemia, and hypertension [34, 35]. These risk factors are common in diabetes, but data suggest that diabetes is an independent risk factor for CVD.

Patients with diabetes, especially T2DM, have many traditional risk factors for CVD, including central obesity, dyslipidemia, and hypertension [36]. In general this combination is referred to as “metabolic syndrome” (central adiposity, dyslipidemia, hyperglycemia, and hypertension) [37]. Along with the independent risk factor of diabetes, these factors can act both independently and cumulatively over time to significantly increase the risk for CVD. The combination of hyperglycemia, insulin resistance, dyslipidemia, hypertension, and chronic inflammation can injure the vascular endothelium, leading to macrovasculopathy and CVD in patients with T2DM [38].

In the Diabetes Control and Complications Trial (DCCT), patients who received the conventional therapy for 6.5 years were compared to the group of patients who received the intensive insulin therapy. The intensive insulin therapy group showed a reduction in serious cardiovascular events, including cardiovascular death [39]. In the report from DCCT/EDIC, reporting the follow-up of 1429 patients over a period of 27 years, there was a large reduction in all-cause mortality in patients initially assigned to intensive therapy (43 deaths in the intensive therapy group versus 64 in the conventional group) [40]. The most common causes of death were CVD (22.4%), cancer (19.6%), and acute diabetes complications: hypoglycemia and diabetic ketoacidosis (17.8%), and accidents or suicide (19.6%). All-cause mortality was higher in patients with higher mean A1C levels and in those patients with additional renal disease. Surprisingly, the intensive insulin therapy for 6.5 years during the DCCT period reduced the risk of mortality in this group over the next 20 years compared with conventional therapy, despite an absence of a difference in A1C values during the post-DCCT trial period.

Traditionally, diabetes and CVD were limited to Western countries, but it is suggested by recent evidence that these conditions are rapidly emerging in resource-limited regions of the world, and estimates indicate that 80% of people with diabetes worldwide will die from CVD [41, 42].

### Myocardial infarction (MI)

The risk for a first myocardial infarction (MI) in patients with diabetes is five times greater than in a population with similar risk factors but without diabetes. The risk for a recurrent MI is twice as high than people who previously had an MI but who do not have diabetes. These data indicate that the risk for a MI in patients with diabetes but who have not had a MI is similar to that in patients without diabetes but with a previous MI [42]. After sustaining a MI, patients with diabetes have a poorer long-term prognosis, increasing the risk for congestive heart failure and death [43]. Even the population with only insulin resistance (often a prodrome of developing T2DM) have an increased risk for CVD [44].

### Cerebrovascular disease

The presence of diabetes adversely affects cerebrovascular circulation by increasing the risk of intracranial and extracranial atherosclerosis [45]. There is no difference in the prevalence and incidence of hemorrhagic stroke among patients with T2DMcompared to non-diabetic patients. Therefore, the excess risk of stroke is due to the high incidence of ischemic strokes in diabetic patients. People with diabetes have an increased occurrence of traditional risk factors for stroke, including high low-density lipoprotein (LDL) cholesterol, elevated blood pressure, smoking, low high-density lipoprotein (HDL) cholesterol, high levels of total triglycerides, central obesity, heart failure, and atrial fibrillation [46]. However, after these factors are controlled for, diabetes remains a strong predictor for stroke, suggesting that the presence of diabetes carries an additional and independent risk for stroke [47]. Besides being an independent risk factor for stroke, diabetes is also a risk factor for sudden and eventual death from stroke [47, 48]. After suffering from a stroke, patients with diabetes show more severe neurological deficits and disability, a poorer long-term prognosis, and a higher incidence of stroke recurrence than people without diabetes [49–53].

Although its precise relationship remains unclear, hyperglycemia and hyperinsulinemia appear to be a significant factor in stroke development [54–56]. Secondly, elevated blood levels of chronic inflammatory markers are associated with an increased risk for stroke [57]. Finally, the concomitant presence of diabetic retinopathy, microalbuminuria, proteinuria, and hyperuricemia are additional factors related to increased stroke risk [58–60].

### Peripheral artery disease

Peripheral artery disease (PAD) is characterized by stenosis and/or occlusion of the lower-extremity arteries [61].

PAD, like the aforementioned vascular diseases, is related to the duration and severity of diabetes [62, 63]. As in other diabetes-related complications, hyperglycemia, specifically glycosylated hemoglobin (HbA1c), appears to be a significant factor in the development of PAD [64]. With every 1% increase in HbA1c, there was a 28% increase in the risk of PAD in the United Kingdom Prospective Diabetes Study (UKPDS) [65].

In the majority of patients, a large single-level disease often manifests initially as claudication. The multilevel disease can also manifest as claudication, provided that sufficient collateral circulation develops. Patients with diabetes are often presented with more advanced diseases and have a worse prognosis. Patients with diabetes are 15 times more likely to have a lower-extremity amputation than patients without diabetes [66].

Besides presenting with more advanced disease, there may also be an anatomic difference in the vascular distribution of PAD that contributes to a worse prognosis. The presence of diabetes is associated with more severe below-the-knee atherosclerosis [62]. Due to the anatomy and the small diameter of the arteries, a location has worse patency after open or endovascular surgery. In patients with diabetes, the anamnesis of physical activity will often identify patients with PAD symptoms and risk factors. However, symptoms of leg pain, the development of ulcers, and functional impairments can be due to PAD, and it can also be a manifestation of diabetic neuropathy (and often both) [62, 63].

For patients with an appropriate history and physical examination, the diagnosis of PAD is established with the measurement of the ankle-brachial index (ABI) [67]. For patients with appropriate symptoms and a normal ABI, an ABI following exercise testing may provide additional information. For the measurement of the ABI, first, the systolic brachial blood pressure measurement at both arms, after which the systolic pressures of the dorsalis pedis and posterior tibial arteries are measured at malleolar level with an 8 MHz Doppler sound in both legs. The ABI is calculated for each leg by dividing the highest systolic ankle pressure by the highest brachial systolic pressure. PAD was defined as a single ABI measurement of less than 0.9 in one or both legs [68].

ABI measurements are both a diagnostic and a prognostic tool [69]. In particular the ABI is very valuable for assessing the progression of PAD and has been reported as an independent marker for cardiac and vascular morbidity and mortality in patients with PAD [69]. The sensivity is lower in patients with diabetes, in particular in presence of peripheral neuropathy. In these cases, other tests have a much higher sensitivity (like toe blood pressure measurements of Doppler waveform analysis). These tests may be able to detect PAD, despite falsely elevated ABI. Besides that, a high ABI is a marker for medial artery calcification and is associated with neuropathy or chronic kidney disease. The earlier mentioned high ABI measurements seem to be linked with a particular form of PAD that is associated with a more diffused atherosclerosis and even microvascular damage. Patients with such a profile need special attention due to the high risk of limb amputation. ABI measurements can be used in patients with diabetes, but need to be interpreted with caution [69]. .Even in the pre-diabetic phase, in the population of patients with dysglycemia, 20% have an abnormal ABI compared with only 7% of patients with normal glucose levels [70].

## Treatment: lifestyle adjustments and risk factor management

There is a consensus that a healthy lifestyle is recommended for the management of diabetes, including the prevention of T2DM [71]. Adapting positive self-care behaviors, such as blood glucose monitoring, a nutrient-rich diet, and exercise are critical to disease progression [72]. Within healthy living, there are three main modifiable risk factors for diabetes: smoking, alcohol, and diet, each of these is discussed in turn.

1. Smoking:

- Smoking is determined as a risk factor for cardiovascular diseases and many more. Despite the extensive body of literature the exact mechanism and pathophysiological link between smoking, diabetes and glucose homeostasis is still not fully understood [73, 74]. Evidence shows that smoking increases the risk of diabetes and mortality [75, 76] and has a negative effect on the common conditions associated with diabetes. Studies have shown that smoking increases insulin resistance and negatively affects glucose control [77].

Smoking has an impact on microvascular complications such as nephropathy, retinopathy, and neuropathy. Evidence shows that smoking can increase the risk of both incidence and progression of neuropathy, especially with T1DM [74], and a meta-analysis of 19 observational studies showed an increased risk of neuropathy in smokers with T1DM and T2DM.

However, a meta-analysis of 73 studies found that the risk of diabetic retinopathy increased in T1D but significantly decreased in T2DM [78].

Neuropathy is one of the most common conditions associated with diabetic foot ulcers, with 78% of people presenting with neuropathy [26]. Smoking may exacerbate diabetic neuropathy partly through the mechanism of oxidative stress, leading to cellular damage and apoptosis (XIA). Other than glycemic control, there is no cure for neuropathy, and with the risks of poor wound healing and evidence of increased foot amputations as a result of Diabetes [79], smoking can have a negative impact on quality of life for people with diabetes.

Macrovascular complications include an increase in coronary heart disease compared with non-smoking counterparts, up to 4 times greater risk in Type 1 and T2DM [80]. A systematic review and meta-analysis with diabetic smokers and risk of cardiovascular events included 48 studies on smoking and risk of total mortality, 13 on cardiovascular mortality, 16 on total cardiovascular disease, 21 on coronary heart disease, 15 on stroke, 3 on peripheral artery disease and 4 on heart failure. Results showed an adjusted risk ratio (RR) associated with smoking of 1.55 for total mortality and 1.49 for cardiovascular mortality. For patients with diabetes, smoking increased the pooled RR for total cardiovascular disease as 1.44, coronary heart disease (CHD) as 1.51, stroke as 1.54, and heart failure as 1.43. The risk of PAD was more than double in patients with diabetes who smoke at RR = 2.15 [81]. Evidence shows that smoking cessation demonstrates clear benefits in reducing or slowing the risk for cardiovascular morbidity and mortality in people with diabetes as it does for the general population [82].

2. Alcohol:

Alcohol (ethanol) is a risk factor for hypoglycemia in T1DM, and a systematic review of 13 studies showed consistent recommendations for alcohol to only be consumed alongside food intake [83]. With T2DM, studies show that moderate alcohol consumption may be protective, reducing the risk of cardiovascular disease and mortality [84], decreasing the incidence of diabetes in many studies, but heavy and/or binge drinkers are at increased risk for diabetes; the type of alcohol, sex, and body mass index (BMI) can additionally affect outcomes [85].

However, alcohol use may detrimentally impact positive health behaviors. Alcohol consumption may lead to decreased compliance with diet, medication, exercise, and glucose self-monitoring, regardless of the amount of alcohol consumed [72]. Given extant evidence that moderate alcohol intake may have cardiovascular benefits for patients with diabetes, a balanced examination of trade-offs between cardiovascular benefits against the potential risk of lower adherence with self-care behaviors warrants further investigation [86].

3. Diet:

Diet has always been central to understanding diabetes, with historical accounts of symptoms attributed to the disease related to food consumption such as sweets and carbohydrates [2]. The goal of diet in diabetes (type 1 and 2) is to decrease the risk of diabetes and CVD by promoting healthy food choices. The American Diabetes Association (ADA) recommends the role of diet to achieve and maintain blood glucose levels in as normal a range as possible, lipid and lipoprotein profile that reduces the risk of vascular disease and regulated blood pressure while preventing/slowing the rate of complications of diabetes [87].

Adhering to the recommended amounts of macro and micronutrients of carbohydrates, fat, protein, vitamins, and minerals form part of the underlying mechanisms needed to regulate glucose metabolism to manage diabetes effectively [88], whether alone or in combination with drug therapy. Studies have shown a positive association between diets high in sugar and the development of T2DM [89], and an increase in the risk of insulin resistance and T2DM was found in people with high intakes of red meat, sweets, and fried food [90]. A systematic review of low carbohydrate, fasting, macrobiotic, Mediterranean, vegetarian, and vegan diets showed that the latter three offered better glycaemic control in individuals with T2DM [91].

## Treatment: medication and bariatric and metabolic surgery (BMS)

In case lifestyle adjustments are not giving satisfactory results, the second line of treatment is usually medication, particularly metformin and/or sulfonylureas (in the case of T2DM). In the case of T1DM, the preferred treatment is insulin replacement [6, 7, 10]. With the changing landscape of pharmacotherapy (for example, GLP-1 agonists) and the increasing understanding of the physiology of diabetes remission after BMS, the physiology became even more complex, and we might need a diabetes re-classification. To understand this, we will discuss the trials assessing clinical outcomes of BMS in patients with T2DM [6, 7, 10].

The prevalence of diabetes is increasing worldwide, and most of the cases are T2DM. The relationship between T2DM and obesity is well established, and surgical treatment is widely used for patients with obesity with T2DM. T2DM is associated with obesity and multiple metabolic derangements, leading to increased morbidity, mortality, and financial burden. Randomized controlled trials (RCT) demonstrate that BS is considered the most effective treatment for obesity treatment by weight loss and comorbidity resolution and has recently shown the efficacy and superiority of surgery over to the best medical therapy alone, achieving improvement hyperglycemia [92, 93]. Table 2 shows the most recent level 1 evidence indicating the long-term efficacy of BS in the remission of T2DM compared to medical therapy.

**Table 2:** Recent level 1 evidence confirming the superiority of bariatric surgery (BS) over medical therapy as a treatment of type 2 diabetes mellitus (T2DM)

Khorgami et al. 2019 [94]	Meta-analysis of 7 RCTs found that the chance of remission of T2DM was significantly higher after BS compared with medical management after at least 2-year follow-up (risk ratio (RR) = 10, 95% CI 5.5-17.9, *p* < 0.001).
Sharples & Mahawar 2020 [95]	A meta-analysis examining 5-year outcomes reported that the resolution of T2DM was 37.4 and 27.5% after RYGB and SG respectively.
Mingrone et al. 2021 [96]	RCT showed the 10-year remission rates for T2DM were significantly higher in the surgical group (BPD 50%, RYGB 25%, medical therapy 5.5%)

*Abbreviations*: *RCT* Randomised controlled trial, *T2DM* type 2 diabetes mellitus, *BS* bariatric surgery, *RYGB* Roux en Y Gastric Bypass, *SG* Sleeve Gastrectomy, *BPD* Biliopancreatic diversion

The mechanisms seem to extend beyond the magnitude of weight loss alone and include improvements in incretin profiles, insulin secretion, and insulin sensitivity. MS offers similar benefits in individuals with BMI 30–35 kg/m2, compared with those with higher BMI. There is a better understanding of gut hormones and nonhormonal factors on weight loss and glucose metabolism.

A five-year follow-up analysis of STAMPEDE (Surgical Treatment and Medications Potentially Eradicate Diabetes Efficiently) trial was among the first to provide level I evidence on the efficacy of BS in T2DM remission and control [93]. Long-term data from the Swedish Obese Subjects (SOS) study [97] suggest that T2DM remission decreases over time. However, this observation is based mostly on results with a procedure that is no longer performed.

A wide variety of BMS procedures initially designed to promote weight loss have been found to powerfully treat T2DM, causing remission in most cases, through diverse mechanisms additional to the secondary consequences of weight loss. The Fifth IFSO Global Registry Report reported that almost 833,687 metabolic procedures were performed worldwide in 2019 [98], of them Sleeve gastrectomy (SG) remained the most commonly performed bariatric procedure (*N* = 305,242; 58.6%) followed by Roux-en-Y gastric bypass (RYGB) (*N* = 162,613; 31.2%), One anastomosis gastric bypass (OAGB) (*N* = 21,613; 4.1%), Gastric band (AGB) (*N* = 19,255; 3.7%), and Duodenal switch with sleeve (*N* = 2554; 0.5%).

BS is currently (according to national and international guidelines) only advised in patients with T2DM with a BMI ≥35 kg/m^2^. This somewhat outdated evidence is being challenged by several studies that indicate that patient a lower BMI and T2DM can also benefit from MS. A recent meta-analysis showed that diabetes remission is comparable between patients with a BMI ≥35 kg/m^2^ and patients with a BMI < 35 kg/m^2^ (71% vs. 72%, respectively) [99].

However, AGB, SG, SADI-S (Single-Anastomosis Duodenal Switch), RYGB, and OAGB incorporate different surgical approaches and differential effects on metabolic outcomes.

RYGB and SG are the most commonly performed bariatric surgical procedures [100–102] and result in significant and sustained weight loss accompanied by dramatic glucose metabolism changes [103, 104]. However, surgeons continue to explore other procedures that may carry advantages in surgical or metabolic improvements. Among these procedures are the OAGB and the SADI-S, both of which produce significant improvements in body weight and glucose homeostasis [105–107].

Commonly speaking, surgical procedures with intestinal diversion and/or duodenal-jejunal exclusion have consistently shown beneficial effects on glucose homeostasis by reducing insulin resistance and increasing insulin secretion [108]. A recent meta-analysis reported an overall remission rate of 78.1% for diabetic patients undergoing BS [102, 109].

A RCT found that OAGB produced weight loss comparative to SG [110], although OAGB was associated with better glycaemic control. A recent report on the weight outcomes of SADI-S vs. RYGB indicated no differences in weight loss or diabetes remission [111–113]. There is now enough evidence to state that BS reduces mortality in patients with diabetes. In the analysis by Adams et al. [114], deaths attributed to diabetes were reduced by 92%.

### Laparoscopic adjustable gastric banding (LAGB)

A dramatic metabolic improvement associated with frequent complete remission of recent-onset T2DM has recently been reported 2 years after LAGB [98, 115, 116]. A meta-analysis [109] reported an overall remission of diabetes undergoing BS. However, not all bariatric procedures were equally effective. Instead, a steady trend of increasing efficacy correlated with the degree of weight loss, from gastric banding at the lower end of the spectrum to biliopancreatic diversion and duodenal switch yielding the greatest results. In a single-institution study [117] of the 5-year outcomes after LAGB, we found a 40% rate of remission and a 72% improvement rate. Although such results were not as positive as those from the studies of RYGB, they nonetheless represent a substantial benefit compared with non-operative traditional diabetes management. Despite the significant improvement in individual metabolic parameters, 73% of patients remained diabetic at 5 years in this intention-to-treat analysis. Moreover, nine (41%) of the 22 diabetic patients failed to meet the 7% HbA1c target level promoted by the ADA [118].

### Roux-en-Y gastric bypass (RYGB)

Two large case-series studies, by Pories et al. [119] and Schauer et al. [120], focused principally on diabetes outcomes after RYGB. In the former study, mean fasting blood glucose (FBG) decreased from clearly diabetic values to near-normal levels (117 mg%), and HbA1c fell to normal levels (6.6%) without diabetes medicines in 89% of patients. In the latest study by Schauer et al. [92], researchers provided an in-depth evaluation of the clinical outcome in 240 patients with morbid obesity and diabetes with a follow-up rate of 80%.

It was shown that there was a BMI decrease from 50.1 kg/m^2^ to 34 kg/m^2^ with a mean concomitant excess weight loss of 60%. This resulted in complete remission of the T2DM in 83% of the patients (normale fasting plasma glucose and HbA1c concentrations) or a significant improvement in 17% of the patients. Both also resulted in a significant decrease in usage of oral antidiabetic agents (80%) and insulin (79%). followed surgical treatment. Patients with the shortest duration (< 5 years), the mildest form of type 2 diabetes (diet controlled), and the greatest weight loss after surgery were most likely to achieve complete resolution of T2DM [92, 93].

The multi-center SOS study compared BS (LAGB, *n* = 156; VBG, *n* = 451; RYGB, *n* = 34) with medical weight-loss treatment in well-matched obese patients [121]. BS caused an average 16.1% weight loss at 10 years, compared with a small weight gain in control subjects. Mean weight loss was greater after RYGB (− 25.0 kg) than after LAGB (− 13.2 kg) or VBG (− 16.5 kg). Mean FBG tended to increase during the study in non-surgical controls (+ 18.7% at 10 years), whereas a substantial decrease was seen in surgical patients at 2 years (− 13.6%) and 10 years (− 2.5%). The risk of diabetes was more than three times lower for surgically treated patients at 10 years, and recovery rates from diabetes were three times greater [121].

### Sleeve gastrectomy (SG)

As SG gained popularity, this procedure’s effectiveness on weight loss and diabetes remission began to be scrutinized [122]. Studies have compared the effectiveness of SG to RYGB in terms of diabetes remission and found comparable results. A study comparing metabolic syndrome of severely obese T2DM subjects following SG and RYGB surgery found equivalent resolution rates, with 84.6% of both cohorts achieving resolution of T2DM at 1 year (*p* = 0.618) [123]. Another study comparing RYGB and SG in patients with obesity found a remission rate of 22 and 21.5%, respectively, 1 and 2 years post-surgery [124].

### One anastomosis gastric bypass – mini gastric bypass (OAGB-MGB)

In terms of metabolic efficacy related to T2DM, OAGB MGB seems to give similar (or even better results) than SG and RYGB. In a systematic review done by Parmar and colleagues it was seen that the T2DM and hypertension remission rates were 83.7 and 66.9% respectively [125].

Quan Y et al. [126] performed a systematic review comparing the efficacy of laparoscopic OAGB-MGB for obesity and T2DM with other bariatric procedures. This review included 6 studies comparing OAGB-MGB with LSG. They reported a significantly higher T2DM remission rate in OAGB-MGB compared with LSG. Also, OAGB-MGB had a significantly lower revision rate. In Quan et al.’s systematic review of 5 studies comparing outcomes of OAGB-MGB and RYGB, they reported that OAGB-MGB had significantly better %EWL and remission of T2DM and had fewer complications [126].

Lee et al. [111] reported more than 80% resolution of metabolic syndrome in their retrospective series of 1163 patients compared to RYGB. More recently, the YOMEGA RCT [127] showed that 60% of diabetic patients achieved complete remission with OAGB-MGB compared with 38% with RYGB. Partial remission rates were 10 and 6%, respectively.

Recently, Sjöström et al. reported that in patients with obesity and T2DM from the SOS trial, a 2-cohort prospective observational study with long-term follow-up, BMS was more frequently associated with T2DM remission and fewer micro and macrovascular complications than usual non-surgical care [97]. These findings were identified despite most patients having undergone purely restrictive procedures such as the vertical banded gastroplasty, which have not been shown to have weight-independent metabolic changes.

## Do we need a diabetes re-classification?

Bariatric surgery is increasingly being proposed as a treatment option for obesity and T2DM because of failure of medical management. Many surgical techniques exist which modulate different aspects of gastrointestinal physiology and will result in weight loss and remission of comorbidities [102, 128]. However, with so many physiological modulators in place, such as the gastrointestinal hormones like GLP-1 and ghrelin, PYY, oxyntomodulin, creating a new classification for the spectrum of diabetes and related disease is a major yet very difficult task.

In terms of mechanistic aspects of BMS, there is a major distinction between bariatric procedures that rely on restriction, malabsorption, or a combination of both [101, 102]. Weight loss and glycaemic effects were traditionally thought to be results of caloric restriction and/or malabsorption of ingested nutrients. The systematic review done by Panunzi et al. [99] showed that diabetes remission rates were similar in patients with a BMI > 35 kg/m^2^ compared with patients with a BMI < 35 kg m^2^. Also the baseline BMI did not have any effect on diabetes remission rates [99]. Still, more recent studies have demonstrated that changes in the physiology of energy balance and body fat mass are the primary mechanisms. Indeed, widespread alterations in the secretion and activity of hormones and neurotransmitters affecting appetite, satiety, energy expenditure, and glucose metabolism in response to these surgical procedures have been increasingly recognized [109, 129].

In a meta-analysis and systematic review of existing RCTs directly comparing various surgical vs. non-surgical treatment for diabetes, BS was associated with greater weight loss, higher remission rates of T2DM and metabolic syndrome, better lipid profiles, greater improvement in the quality of life, and substantial reductions in medication requirements [130, 131]. Mechanistic evidence further suggests that the duodenum and jejunum (proximal gut) bypass or exclusion may directly benefit glycemic control beyond those mediated by weight loss [132].

Buchwald et al. [102] reported that complete resolution of T2DM (defined as discontinuation of all diabetes-related medications and blood glucose levels within the normal range) occurred in 78.1% of cases after BS. This percentage increased to 86.6% when counting patients reporting improved glycemic control, and diabetes resolution occurred in concomitance with an average weight loss of 38.5 kg (55.9% of the excess weight) [102]. However, Panunzi et al. [99] reported that diabetes remission after BMS (in 94,579 surgical patients) was independent of the mean baseline BMI (≥35 kg/m^2^ compared with < 35 kg/m^2^. The diabetes remission rates were similar (71% vs. 72%, respectively) [99].

When looking at an updated diabetes classification, we need to take into account the different modes of action of the gastrointestinal hormones [133]. As scientists, we are tempted to correlate the effects of BS with the change of the gastrointestinal hormones postoperatively [134]. In the remission of T2DM after BS, there is now intriguing evidence that GLP-1 receptor agonists are not able to promote remission of T2DM as surgical procedures do. They are effective agents in the treatment of T2DM, and some even consider them as BS ‘mimetics’ because of the improved glycaemic control and weight loss during therapy [134–137]. Unfortunately, this is not the case [138].

## Conclusion

Diabetes Mellitus is a complex multifactorial disease, leading to high morbidity and mortality. With the development of newer drugs and improved surgical options, our knowledge of diabetes and its physiology increased incrementally. However, with this increase in knowledge, we are still not able to fully understand its pathophysiology and therefore based on the current literature a new reclassification is difficult to made. Future research will hopefully guide clinicians to optimal medical and/or surgical treatment for diabetes and provide further structure in a potential reclassification of diabetes.

## Data Availability

Upon request, please contact the corresponding author (dr. S. Pouwels).
